# The high prevalence of *Clostridioides difficile* among nursing home elders associates with a dysbiotic microbiome

**DOI:** 10.1080/19490976.2021.1897209

**Published:** 2021-03-25

**Authors:** John P. Haran, Doyle V. Ward, Shakti K. Bhattarai, Ethan Loew, Protiva Dutta, Amanda Higgins, Beth A. McCormick, Vanni Bucci

**Affiliations:** a Department of Emergency Medicine, University of Massachusetts Medical School, Worcester, MA, USA; b Department of Microbiology and Physiological Systems, University of Massachusetts Medical School, Worcester, MA, USA; c Program in Microbiome Dynamics, University of Massachusetts Medical School, Worcester, MA, USA

**Keywords:** *Clostridioides difficile* colonization, nursing home elders, gut microbiome, proton pump inhibitor, dysbiosis, bile acids

## Abstract

*Clostridioides difficile* disproportionally affects the elderly living in nursing homes (NHs). Our objective was to explore the prevalence of *C. difficile* in NH elders, over time and to determine whether the microbiome or other clinical factors are associated with *C. difficile* colonization.

We collected serial stool samples from NH residents. *C. difficile* prevalence was determined by quantitative polymerase-chain reaction detection of Toxin genes *tcdA* and *tcdB*; microbiome composition was determined by shotgun metagenomic sequencing. We used mixed-effect random forest modeling machine to determine bacterial taxa whose abundance is associated with *C. difficile* prevalence while controlling for clinical covariates including demographics, medications, and past medical history.

We enrolled 167 NH elders who contributed 506 stool samples. Of the 123 elders providing multiple samples, 30 (24.4%) elders yielded multiple samples in which *C. difficile* was detected and 78 (46.7%) had at least one *C. difficile* positive sample. Elders with *C. difficile* positive samples were characterized by increased abundances of pathogenic or inflammatory-associated bacterial taxa and by lower abundances of taxa with anti-inflammatory or symbiotic properties. Proton pump inhibitor (PPI) use is associated with lower prevalence of *C. difficile* (Odds Ratio 0.46; 95%CI, 0.22–0.99) and the abundance of bacterial species with known beneficial effects was higher in PPI users and markedly lower in elders with high *C. difficile* prevalence.

*C. difficile* is prevalent among NH elders and a dysbiotic gut microbiome associates with *C. difficile* colonization status. Manipulating the gut microbiome may prove to be a key strategy in the reduction of *C. difficile* in the NH.

## Introduction

*Clostridioides difficile* infection (CDI), the leading cause of gastroenterologic hospitalizations and associated deaths,^1^ remains at a historically high level with hospital stays from the disease tripling over the past decade.^2^ The elderly are disproportionally affected^3,4^ with the rate of CDI being several fold higher in individuals 65 years of age and older^5^ and an increased risk of 2% for each additional year after 65 years.^6^ Not only are elders at increased risk of acquiring CDI but they also have higher rates of recurrence, complications, and death.^7^ Elders living in nursing homes (NHs) are now the predominant group suffering from CDI.^3,8^ On average, 40% to 50% of new CDI cases come from elders living in nursing homes.^9,10^ Most NHs in the US have structured infection control and prevention programs,^11^ however environmental measures to control CDI, such as enforcing hand hygiene, contact precautions, and decontamination procedures are employed only after CDI is identified. These measures have not been able to stem CDI concerns in NHs.^12^

One factor that has become of increasing contemporary interest and a target of preventive strategies is the human gut microbiome. The microbiome is a vast ecosystem of microbes that can influence human health and disease.^13^ The intestinal flora changes with age, especially as the presence of anaerobes decreases.^14,15^ Elders from nursing homes differ from community-dwelling counterparts as their microbiome composition has a higher proportions of the phylum *Bacteroidetes* and decreased abundances of other bacteria beneficial to human health, at the genus levels.^16,17^ Dysbiosis is a term describing a microbial imbalance or maladaptation on or inside the body and can be defined as either the loss or gain of bacteria which promote health or disease.^18,19^ The microbiome of nursing home elders forms a dysbiotic pattern with increasing age, frailty, and malnutrition scores.^20^ These microbial dysbiosis patterns have associations with different disease states, however connecting these NH dysbiotic patterns to *C. difficile* colonization and attempting to correct them may serve as a means to prevent disease spread. Another target for CDI prevention is reducing the prevalence of *C. difficile* in the environment. The prevalence of *C. difficile* in stool is the highest among those living in nursing homes with 20% to 50% of residents affected, compared to 1.6% in the general community and 9.5% in the outpatient setting.^14,21^ Higher prevalence of asymptomatic colonization with *C. difficile* is a well-documented source of new CDI cases with spread of the bacteria to vulnerable individuals, however approaches to managing colonization as a means to prevent CDI are lacking.^22–24^

Currently, there is a lack of knowledge concerning the risk factors contributing to *C. difficile* colonization in NH elders. A better understanding of how clinical factors and microbiome composition contribute to *C. difficile* colonization in NH elder would provide a novel tool for preventing CDI. Accordingly, we set out to follow longitudinally a cohort of elders from multiple NH facilities to investigate: 1) the patterns of *C. difficile* colonization; 2) the associations of *C. difficile* colonization with medication exposures and other clinical variables; and 3) the associations of *C. difficile* colonization with characteristics of the elder gut microbiome. Our findings contribute to the understanding of microbiome composition as a potential target to reduce CDI burden in the elderly.

## Results

### Characteristics of the study subjects

Over a 3-year period, we enrolled and followed 167 NH elders from 5 different facilities collecting monthly samples, totaling 506 clinical samples. Forty-four (26.35%) of the residents enrolled provided only a single stool sample for the following reasons: 23 (52.3%) elders were on a floor where multiple sample collection did not occur, 12 (27.3%) residents chose to withdraw from the study, 5 (11.4%) died after the first sample collection, 3 (6.8%) had their nurse stop collection due to social issues and, 1 (2.3%) moved out of the facility. Of the remaining, we had an average of 3.7 samples collected per elder. No residents included in this analysis were exposed to antimicrobials nor were hospitalized during the study period. The average participant age was 85.2 years (SD 9.1) of which 18.1% were male, 40 (23.9%) were hospitalized in the past year, and 31 (18.7%) had an antimicrobial exposure in the 6-months preceding enrollment. Both frailty and malnutrition were prevalent with an average clinical frailty score of 6.5 (s = 1.0) or halfway between moderate and severely frail, and a malnutrition indicator categorical score of 2.1 (s = 0.7). Of note, no residents experienced diarrheal symptoms nor were diagnosed with CDI during the course of this study.

**A majority of NH elders have detectable *C. difficile* with one quarter of elders colonized over multiple timepoints**

**Figure 1.**
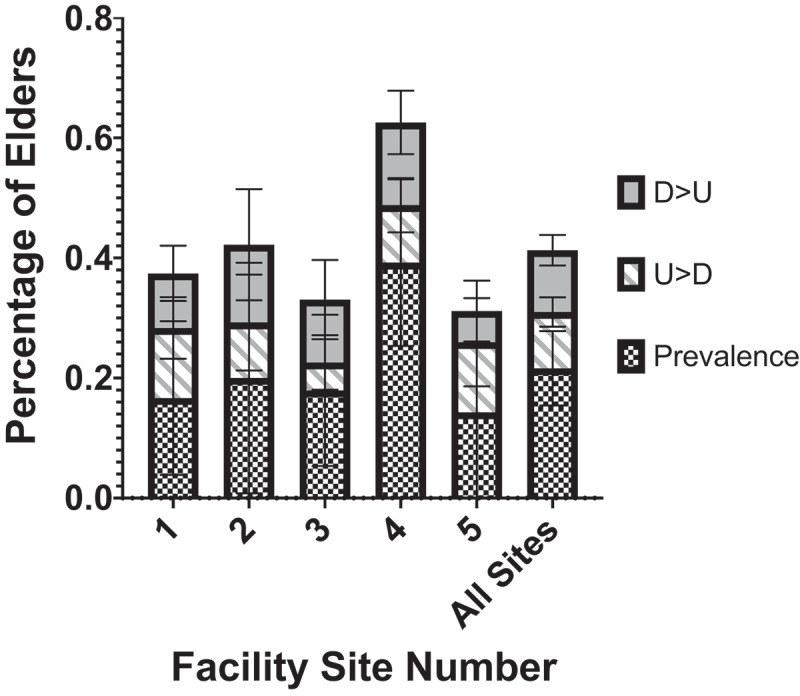
Mean percentage of initial samples testing positive for *C. difficile* (prevalence) by nursing home facility site and subsequent samples that changed from undetectable to detectable (U > D) and detectable to undetectable (D > U). This data represents the percentage from the initial and all subsequent patient samples where the following sample changed status. Error bars depict 95% confidence intervals

Of the 506 samples collected 122 (24.1%) were positive for *C. difficile*. Over the course of the study, 78 (46.7%) elders had at least one time point in which *C. difficile* was detected in their stool. Across all five sites, the prevalence of *C. difficile* from the first elder stool sample taken was 24.0%. For elders with serial sampling, 52 (9.7%) elders had *C. difficile* positive samples following a previous *C. difficile* negative sample; 57 (10.6%) elders had *C. difficile* negative samples following a previous *C. difficile* positive sample. On average there was just over 1 month between sampling timepoints (48.8 days, 38.9 sd). For elders that went from undetectable to detectable (U > D) there were on average 51.1 days and 40.0 days from detectable to undetectable (D > U) elders. Among elders with multiple positive samples, the average time difference between positive samples was 42.6 days (sd 25.0). There was no significant difference between the five sites in the percent of serial sampling that changed the *C. difficile* status. Site 4, however, had a higher prevalence of *C. difficile* (*p* = .048), as determined from first samples collected per elder (Figure 1).

Of the 123 elders with multiple samples, there were 41 (33.3%) with a single positive sample and 30 (24.4%) with multiple samples positive for *C. difficile*. The number and percentage of residents colonized by *C. difficile* did not vary significantly by the nursing home facility nor floor/wing in which the elder lived. We did not observe any significant differences in resident demographics nor clinical scores (both frailty and malnutrition) between colonized and non-colonized residents (Table 1). The only medication significantly associated with *C. difficile* colonization (single and multiple) was proton pump inhibitors (Table 1). Remarkably, elders taking a PPI daily were 63% less likely to be colonized with *C. difficile* (OR 0.37, 95%CI 0.17,0.82). No other medication showed any significant associations and the only medical condition associated with colonization was a history of a cerebrovascular accident (CVA). Residents colonized with *C. difficile* had a non-statistically significant higher percentage of an antibiotic exposure within the preceding 6 months.

**Table 1.** Clinical characteristics among residents that were colonized with *clostridium difficile* at one time point, multiple time points, or none

	None (52)	One (41)	Multiple (30)	p-value
Demographics				
Age (mean [SD]) (yr)	84.6 (10.2)	86.3 (9.7)	87.3 (6.5)	0.40
Gender Male	11 (21.1)	6 (14.6)	2 (6.7)	0.21
Clinical Frailty Score (mean [SD])	6.4 (1.0)	6.7 (0.4)	6.3 1.0)	0.50
Malnutrition (mean [SD])	2.1 (0.7)	2.1 (0.7)	1.9 (0.7)	0.78
CCI Score (mean [SD])	0.8 (1.7)	0.8 (1.7)	1.1 (1.9)	0.74
Polypharmacy	24 (46.2)	13 (31.7)	14 (46.7)	0.30
Antibiotic Exposure	6 (11.5)	11 (26.8)	7 (23.3)	0.15
Hospital Exposure	16 (30.8)	9 (22.0)	5 (16.7)	0.33
Medications (only those with *p* < .10)				
PPI	22 (42.3)	7 (17.5)	8 (26.7)	0.033
Other Medications in Model				
GABA Analogs	6 (11.5)	5 (12.5)	7 (23.3)	0.31
Benzodiazepines	6 (11.5)	4 (10.0)	7 (23.3)	0.23
Valproic Acid	0 (0.0)	2 (5.0)	1 (3.3)	0.29
Thiazide diuretics	0 (0.0)	1 (2.5)	1 (3.3)	0.45
Antiplatelet Medications	4 (7.7)	2 (5.0)	0 (0.0)	0.30
Oral Medications for Diabetes	3 (5.8)	2 (5.0)	0 (0.0)	0.42
Past Medical History				
Alzheimer’s Disease	12 (23.1)	14 (34.1)	4 (13.3)	0.13
CVA mild	6 (11.5)	1 (2.5)	6 (20.0)	0.061
CVA severe	0 (0.0)	0 (0.0)	2 (6.7)	0.044
Data presented in number (%) unless otherwise specified. CCI, Charlson comorbidity index. CVA, cerebrovascular accident. PPI, proton pump inhibitor. Polypharmacy is 8 or more daily medications. We used chi-square tests to compare categoric variables and the Student’s t test for continuous variables.				

### *Dysbiotic microbiome profile associates with* clostridioides difficile *colonization*

Using longitudinal shotgun metagenomic sequencing reads from our NH elders profiled for microbial species abundances with Metaphlan2^25^ in combination with demographic, clinical, and medication data we sought to investigate what microbiome and clinical features associate with higher rates of *C. difficile* colonization in the NH environment. We identified a total of 789 species, and after applying a 10% prevalence cutoff we ended up with 218 which we used for the remainder of the analysis. We started by applying unsupervised learning methods, such as correspondence analysis and t-Distributed Stochastic Neighbor Embedding (t-SNE), and, as expected, found that interindividual variability overwhelmingly accounted for the majority of the information in the data (Supplemental Figure 1). Therefore, in order to identify microbiome features that are associated with *C. difficile* colonization, while also accounting for patient-specific effects, we chose to apply mixed-effect random-forest regression.^26,27^ This modeling approach has significant advantages compared to traditional multi-linear regression techniques, as it is agnostic to model structure (e.g. non-parametric regression), it does not need to meet common assumptions underlying classical regression techniques and is able to intrinsically perform ranked feature selection. We modeled the relative abundance of every species as a function of patient’s sex, age, PPI (yes/no), antibiotic in the last 6 months (yes/no), GABA Analogs (yes/no), Benzodiazepines (yes/no), Valproic Acid (yes/no), Thiazide diuretics (yes/no), Antiplatelet Medications (yes/no), Oral Medications for Diabetes (yes/no), and *C. difficile* prevalence (Never, One time Point, Multiple Time points, as 0, 1, 2). Because PPI was found to associate with lower *C. difficile* colonization (Table 1), we hypothesized that this could be microbiome-mediated and hence included in the modeling a *C. difficile*-PPI interaction term. Finally, the patient identifier was used as a random effect. The final model that we trained to data was therefore of the form $X_{s,i\;}=\;f\left(CC\right)+1|\;ID$, where X is the relative abundance of species s in sample i, f is a general non-linear function (the random forest) applied to the Clinical Covariates (CC) as fixed effects and 1|ID corresponds to the random effect to account for multiple samples from the same patient. The advantage of this approach is that it allows training using multiple samples from the same patients without needing to average them out. We used permutated importance analysis to determine significance of covariates in predicting microbe abundances.^28^

**Figure 2.**
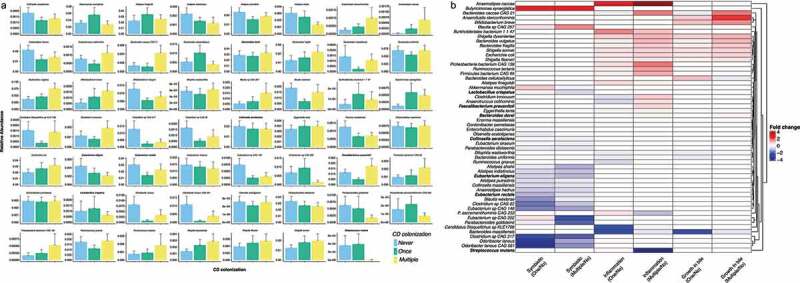
Results from mixed-effect random forest modeling identifies bacteria significantly associated (BH adjusted <0.05) with C. difficile colonization in NH elders. (a) We report barplots showing the average ± standard deviation of the abundance of CD-colonization significantly associated microbes in patients never, once or with multiple samples colonized by C. difficile. The average was calculated from each patient average across multiple microbiome samples. Averaging across patient is only for visualization purpose as the mixed-effect random forest modeling explicitly considered each sample from every patient. (b) heatmap displaying the log fold change between the average abundance in patients once or multi-colonized over those never colonized by C. difficile. Heatmap shows that bacteria with known symbiotic or health-associated properties (including SCFA-producers from the clostridium cluster IV an XIVa) are reduced at higher C. difficile colonization. Oppositely, bacteria associated to gastrointestinal dysbiotic conditions are enriched in elders with higher C. difficile prevalence. Red color indicates fold change increase in C. difficile colonization status while blue is decrease. Species bolded are otUs that overlap with Figure 3

From the modeling, we identified 55 different bacterial species whose abundances were significantly associated with *C. difficile* colonization. We categorically stratified *C. difficile* colonization for each patient by the number of positive samples (i.e. Never, Once, and Multiple) (Figure 2a).

There are several patterns that emerge from this analysis. First, bacterial taxa that are beneficial to human health are enriched in elders without *C. difficile*. For example, multiple butyrate-producing organisms in the *Eubacterium* genus^29^ along with taxa such as *Odoribacter laneus, Alistipes shahii*, and *Parabacteroides distasonis*^30–32^ have higher relative abundance in elders with no *C. difficile* and progressively lower relative abundance in Once and Multiple colonization groups. When confirmed that butyrate production capacity was reduced at higher prevalence of *C. difficile* colonization by repeating the same modeling analysis but this time predicting the abundance of metabolic pathways inferred by running humann2^33^ on the metagenomic data. Among the 211 pathways significantly associated with *C. difficile* prevalence (*p*-value BH adjusted <0.05 (Supplementary Figure S2), we found that both pyruvate fermentation to butyrate and the superpathway of *clostridium acetobutylicum* acidogenic fermentation were reduced in elders colonized Once or Multiple times with *C. difficile* compared to None.

Second, both inflammatory-type and pathobiont species (bacteria that cause or promote disease only when specific genetic or environmental changes have occurred)^34^ were found to be at higher relative abundance among elders with *C. difficile*. Pathogens such as multiple *Shigella* species, *Escherichia coli*, and *Bacteroides fragilis*,^35–37^ all increased in abundance, progressing from Never to Multiple positive samples. Interestingly, this also coincided with higher abundance of the superpathway of lipopolysaccharide (LPS) biosynthesis in Once and Multiple individuals Supplementary Figure S2) which was recently shown to be mostly contributed by *Bacteroidales* and *Enterobacteriales* microbes^38^ and in an increase in the *E. coli*-derived superpathways of fatty acids and unsaturated fatty acids biosynthesis Supplementary Figure S2). Inflammation-associated bacteria *Bilophila wadsworthia, Bacteroides dorei*, and *Firmicutes bacterium*,^39–41^ also showed increased abundances in elders with multiple *C. difficile* positive samples. Lastly, taxa that are either resistant to or grow with exposure to bile exhibited significantly higher abundances among elders with multiple *C. difficile* positive samples. These include *Alistipes putredinis, Anaerofustis stercorihominis*, as well as *Bacteroides* and *Bifidobacterium* species.^30,36,42,43^ Hierarchical clustering of the fold change abundance, between the Once and Multiple positive samples groups relative to Never positive group, indicates reduction of symbiotic (mutually beneficial host–microbiome relationship), or health-associated taxa and an increase in inflammation-associated taxa, with known ability to grow in presence of primary bile acids, with Multiple *C. difficile* colonization (Figure 2b).

Sixty-six individuals were characterized by having samples both negative and positive for C. difficile presence. We therefore asked if the microbiota profile change in patients where there is a change in *C. difficile* status both in terms of species abundances and overall diversity. We compared Shannon Diversity for samples negative and positive with *C. difficile* using mixed-effect modeling and using patient ID as random effect. We found no difference in diversity between samples positive and negative for *C. difficile* presence (Supplemental Figure 3A). We applied Mixed-Effect Random Forest Classification to predict *C. difficile* sample positivity as a function of microbial abundances. The model identified 11 species significantly associated with *C. difficile* sample positivity, and notably identifies an increase in *Akkermansia muciniphila* and in *B. vulgatus* in *C. difficile* positive samples (Supplemental Figure 3B). Interestingly, higher *A. muciniphila* abundance has also been found in CDI elders compared to control groups.^44^

**Figure 3.**
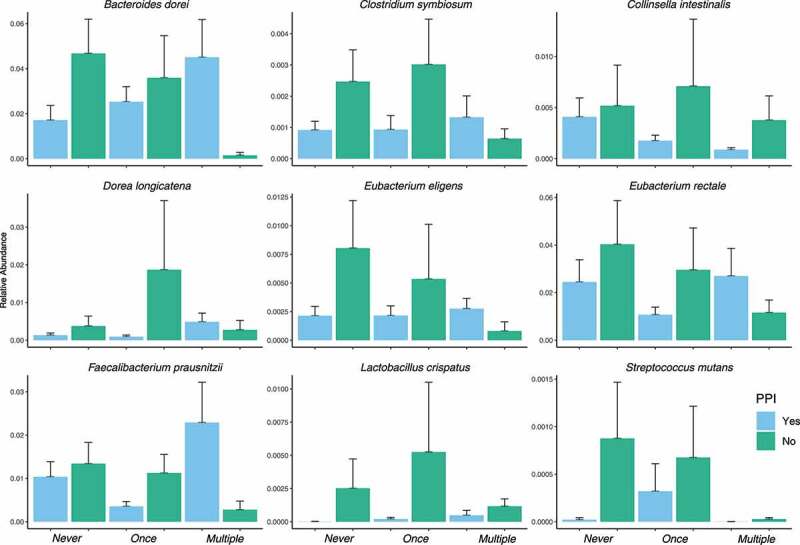
Results from mixed-effect random forest modeling identifies bacteria with significant (BH adjusted < 0.05) C. difficile colonization-PPI interaction terms. The displayed subset are those species that are enriched in PPI = yes in people with no C. difficile detected and that decreased with C. difficile colonization

As we determined that PPI is negatively associated with *C. difficile* prevalence in our bivariate analysis (Table 1), we hypothesized that this protection could be gut microbiota dependent. We therefore investigated the microbiota species that were characterized by having a significant (*p*-value BH adjusted <0.05) *C. difficile*-PPI interaction coefficient from the mixed-effect random forest modeling. From these species, we found that a subset displaying a greater abundance in PPI-treated elders without *C. difficile* compared to PPI-untreated and whose abundance was decreasing with increasing *C. difficile* prevalence. According to these criteria, we identified a set of nine species (Figure 3).

## Discussion

Nursing home elders demonstrated high *C. difficile* prevalence in our study with over half having at least one *C. difficile* positive sample and 1 in 4 elders having multiple positive samples. This colonization pattern suggests, and we further hypothesize, that a subset of elders in the NH may serve as a source of transmission to other individuals. In an attempt to better understand these individuals, we looked for clinical and microbiome associations. Among the clinical, we found daily proton pump inhibitor exposure was associated with a 63% reduced risk of *C. difficile* colonization (i.e. more non-colonized residents were daily PPI users). Reduced abundances of symbiotic or butyrate-producing organisms and higher abundances of pathogenic, inflammatory, or bile acid growing bacterial species were associated with *C. difficile* colonization.

### Clostridium difficile *colonization is common among NH elders*

The prevalence of *C. difficile* colonization observed in our study is consistent with prior reports that have demonstrated a range of colonization in the NH environment with prevalence rates as low as 20% to over 50%.^14,21^ We have expanded upon prior cross-sectional reports by following elders longitudinally. We demonstrate not only a high prevalence but also patterns of colonization where a subset of elders has multiple *C. difficile* samples over time. Elders with multiple positive samples may represent a group of long-term colonized elders who are more likely to serve as reservoirs of *C. difficile* relative to those with only one positive sample, or transiently colonized elders.

There are few studies of *C. difficile* colonization in the elderly, fewer involving elders living in nursing homes. The systematic review noted above did not reveal any association with PPI and *C. difficile* in the elderly.^45^ Among older hospitalized adults, treatment upon admission with PPIs has not been shown to be associated with *C. difficile* colonization.^46^ The use of PPI was also shown not to be associated with *C. difficile* colonization in previous studies with a lower number of subjects, 68 long-term care elders, although a non-statistically significant higher percentage of colonized residents were on a PPI.^21^ Our findings suggest that changes in the intestinal microbiome with acid-reducing medication use may be associated with a less favorable environment for *C. difficile* colonization.

### *A dysbiotic gut microbiome composition associates with* clostridium difficile *colonization*

Elders in our study with Once and Multiple types of *C. difficile* colonization demonstrated a pattern of decreased abundances of symbiotic and/or butyrate-producing organisms and consequent reduction in pathways leading to butyrate production. These elders were found to display increased abundances in pathogenic, inflammatory, or bile acid growing species, along with increased capacity for the production of known inflammatory molecules such as LPS.

Butyrate is a short-chain fatty acid that is known to contribute to the maintenance of the gut barrier functions and has both immunomodulatory and anti-inflammatory properties.^29^ Thus, higher abundances of organisms that produce butyrate are considered to be beneficial. Among patients with CDI, butyrate-producing species have been shown to be depleted,^47^ and butyrate protects against CDI, in mouse models by reducing intestinal inflammation and increasing epithelial tight junctions.^48^ Lack of butyrate-producing species has been linked to many other intestinal disorders such as Crohn’s Disease and colorectal cancer.^29,49^ A lack of butyrate-producing organisms in NH elders may contribute to a favorable environment for *C. difficile* colonization.

Multiple pathogenic bacterial species exhibit increased relative abundance in *C. difficile* colonized elders. These include *B. fragilis*, the most commonly isolated anaerobic pathogen,^36^ *Eggerthella lenta*, a significant human pathogen that is often associated with serious gastric pathology,^50^ *E. coli*, and multiple *Shigella* species, which has been reported to be present during CDI in elderly NH patients.^51,52^ Inflammation-associated species were also present in higher abundances especially among elders with multiple *C. difficile* positive samples. Species such as *Proteobacteria bacterium*, a commonly found species in inflammatory disorders,^53^ *Anaerostipes caccae* which is positively associated with CDI in Irritable Bowel Disease,^54^ *Bacteroides vulgatus*, a species that has recently been identified as influencing neuroinflammatory signaling,^55^ and *Ruminococcus gnavus*, which has been associated with a dysbiotic microbiota.^56^ Taken together, higher abundances of pathogenic and inflammatory bacteria associate with *C. difficile* colonization.

Bile acids, detergent-like biological substances synthesized in the liver from cholesterol, play an important role in the physiology of intestinal bacteria and influence their functionality.^43^ *C. difficile* is dependent upon accessing and modifying endogenous bile salts.^57^ We observed increased abundances of species that grow when exposed to increasing concentrations of bile acids among elders with multiple *C. difficile* positive samples. Several members of the *Bacteroides* genus had higher abundances among *C. difficile* colonized elders. In general, *Bacteroides* is a bile resistant genus while *Bacteroides fragilis*, specifically, is known to play a key role in the enterohepatic circulation of bile acids.^36^ We also found *Bifidobacterium breve* following the same trend as another species that survives in bile.^43^ Interestingly, *Bifidobacterium longum*, which has one of the lowest survival rates in bile among the *Bifidobacterium* genus,^58^ was found at lower abundances among *C. difficile* colonized elders. Other species following this trend of growth in bile and higher abundances in *C. difficile* colonized elders include *Alistipes putredinis*,^59^ *Anaerofustis stercorihominis*,^42^ *Bilophila wadsworthia*,^60^ and the pathogens *Shigella* and *Escherichia coli*.^61^ It is thought that decreases in secondary bile acids may provide a favorable environment in which *C. difficile* can grow and colonize.^62^

**Proton pump use associates with protection against *C. difficile* colonization via commensal gut bacteria**

Proton pump inhibitors have been known for quite some time to be associated with an increased risk of CDI.^6^ A recent large systematic review and meta-analysis including 56 studies involving 366,683 patients demonstrated a two times increased risk of CDI with PPI use.^45^ However, whether this is causation or association is still up for debate given that the exact mechanism of action is unknown.^63,64^ Proton pump inhibitors are one type of medication that changes both overall diversity and the relative abundances of specific bacterial taxa.^65^ Besides diversity, differences among bacterial species composition in the intestines between PPI users and non-users are consistently associated with changes toward a dysbiotic gut microbiome and are in line with known changes in the microbiome that predispose individuals to CDI.^66,67^ With regards to colonization, PPIs have been shown not to promote *C. difficile* colonization in murine models,^68^ and a more recent prospective observational study among older hospitalized adults demonstrated no relationship between PPI use and *C. difficile* colonization.^46^ In other nursing home investigations, the use of PPI had a non-significant inverse relationship with *C. difficile* colonization however among a much smaller cohort.^21^

Here we are reporting on the association of lower risk for *C. difficile* colonization among nursing home elders taking a PPI daily and propose that a possible mechanism lies in the differences in microbiome composition between users and non-users of PPIs. Among non-colonized elders taking a PPI, we noticed enrichment in gut bacteria such as *Eubacterium species* and *Faecalibacterium prausnitzii*. The *Eubacterium spp*. are an important butyrate-producing bacterial species which contributes to the maintenance of the gut barrier functions, and has both immunomodulatory and anti-inflammatory properties.^29^ *Faecalibacterium prausnitzii*, one of the most abundant and important butyrate commensal bacteria of the human gut microbiota,^69,70^ also show enrichment in non-colonized PPI users. The subset of species displaying a greater abundance in PPI-treated elders without *C. difficile* compared to PPI-untreated and whose abundance was decreasing with increasing *C. difficile* prevalence included species five (*C. symbiosum, D. longicatena, E. eligens, E. rectale* and *F. prausnitzii*) belong to the Clostridiales order. These species have also been associated with protection against inflammatory and infectious conditions in both mice and humans, including *C. difficile* infection.^71–73^ Vincent et al. (2013) found that Eubacteria and *Faecalibacterium* are depleted in CDI patients.^73^ It additionally reported a decrease in the family *Bacteroidaceae*, which is consistent with our analysis finding *B. uniformis* enriched in PPI with no CD and reduced with higher prevalence of *C. difficile*, an association noted by others.^74–76^ Our findings suggest that changes in the intestinal microbiome among elders exposed to a PPI may be associated with a less favorable environment for *C. difficile* colonization.

### Strengths and limitations

This study had several notable strengths and limitations. One limitation of this study is that it did not have four stool samples from each resident. This may have led to misclassification of the secondary outcome of multi-colonization in these residents. This is the largest longitudinal cohort of nursing home elders reporting microbiome composition. It is also the largest study to survey NH residents for *C. difficile* colonization. That being said this study is still limited in the number of residents enrolled. A more robust cohort would help us to take a much deeper look at the multiple levels of data and to better explore other classes of medications used less frequently by NH elders. There are potential confounding variables, specifically classes of medications the residents were taking (such as corticosteroids and immunosuppressants) that were not evaluated in this cohort due to the small number of residents on these drugs. Following up this investigation with a cohort including larger numbers of residents from more facilities would strengthen the findings and further explore the dysbiosis associations with medication exposure in the elderly and further address how these dysbiotic patterns are associated with *C. difficile* colonization.

### Conclusions

In conclusion, *C. difficile* colonization is common among NH elders with a large portion of these colonized residents harboring this pathogen over the course of months. *C. difficile* colonization state was associated with an inverse relationship to PPI medication use and key healthy bacterial species are present in elders using a PPI who do not demonstrate colonization. Finally, we found that the abundances of several key intestinal bacterial species were associated with *C. difficile* colonization and thus describe a dysbiotic environment in which *C. difficile* can take residence and thrive. This microbiome is described as having lower abundances of symbiotic or butyrate-producing organisms with increased abundances of organisms that utilize bile acids or are considered pro-inflammatory or pathogenic bacterial species. Further work is needed to see if promoting an elder gut microbiome composition to one that resists colonization could affect the high rates of *C. difficile* colonization seen within the nursing home environment, thus providing a novel approach at preventing this devastating disease.

## Patients and methods

### Study setting and population

This prospective cohort study was approved by the institutional review board at the University of Massachusetts Medical School. This cohort is of NH residents ≥65 years of age who lived in one of five NH facilities in central Massachusetts. We approached residents who had been living in the facility for ≥1 month and did not have any diarrheal illness or antimicrobial exposure within the preceding 4 weeks. Our trained staff used a standardized Capacity for Informed Consent Instrument^77^ that combines capacity assessment questions with observation. If the resident was deemed unable to provide consent, we contacted the healthcare proxy to obtain informed consent. Residents were enrolled for a minimum of 4 months. All residents across the four facilities followed similar low-fiber diets and at each nursing facility, all the elders were fed the same meals that is typical for a nursing home diet. No patients suffered from dysphagia or had a feeding tube.

### Data collection

We conducted baseline and end of study medical record abstraction for factors associated with key study outcomes. These factors included: age, nutritional status, comorbidities, all medications, and frailty.^16^ Prior history of hospitalizations within the past year and antibiotic exposures in the past six months were collected from the medical record. Both daily and as needed medications were obtained from the facility’s medical record. Polypharmacy was defined using the most commonly reported definition of five or more daily medications.^78^ Polypharmacy has been shown to represent a determinant of gut microbiota composition independent of specific drug classes that have detrimental clinical consequences.^79^ We also obtained age, sex, race, and length of NH stay from the medical record. Clinical scoring systems were made from elders during baseline interviews and corroborated from family or facility staff. We used the Charlson Comorbidity Index (CCI) to categorize patients’ medical comorbidities.^80,81^ The CCI is a method of predicting mortality by classifying or weighting comorbid medical conditions and has been widely used to measure the burden of medical diseases.^82^ Frailty was categorized according to the validated and widely utilized Canadian Study of Health and Aging’s (CSHA) 7-point Clinical Frailty Scale (CFS).^83^ This has been previously validated in demonstrating signatures of frailty in the gut microbiota.^20,84,85^ This frailly scale goes from very fit (1) to very severely frail (8). We did not enroll terminally ill patients into this study. We assessed nutritional status using the Mini Nutritional Assessment (MNA) tool.^86–88^ The MNA is a rapid assessment of nutritional status used routinely in elderly NH residents.^88^ Residents were categorized as normal (1), at risk (2), or malnourished (3) based on the MNA survey administered to the residents by trained research staff or the nurse caring for the resident if mentally impaired. All residents enrolled were monitored during their involvement in the study for any changes to their care or for new medication or hospitalization exposures.

### Sample collection, sequence processing, and analysis

DNA was extracted from samples using the PowerMagTM Soil DNA Isolation Kit on an epMotion 5075 TMX liquid handling workstation according to manufacture protocols (MO BIO Laboratories, #27,100-4-EP). Sequencing libraries were constructed using the Nextera XT DNA Library Prep Kit (Illumina, Inc., #FC-131-1096) and sequenced on a NextSeq 500 Sequencing System as 2 × 150 base pair-end reads. Shotgun metagenomic reads were first trimmed and quality filtered to remove sequencing adapters and host contamination using Trimmomatic^89^ and Bowtie2,^90^ respectively, as part of the KneadData pipeline v0.6.1 (https://bitbucket.org/biobakery/kneaddata) using human genome hg19 (GRCh37, GCA_000001405.1). Reads were then profiled for microbial taxonomic abundances using Metaphlan2^91^ and for metabolic pathway abundance using humann2^33^ (as in our previous work).^20,27^ Statistics for the sequencing is provided in Table 2:

**Table 2.** Sequencing statistics

	Tot met	Tot con	R1 met pair	R1 met unpair	R2 met pair	R2 met unpair	R1 con pair	R1 con unpair	R2 con pair	R2 con unpair
Min	11,001	22	5221	173	5221	51	2	0	2	4
1st	1,507,492	1405	676,456	33,390	676,456	11,720	424.5	62	424.5	202
Med	3,631,704	3999	1,599,716	145,184	1,599,716	60,103	1515	247	1515	697
Mean	4,882,975	38,199	2,226,512	262,996	2,226,512	166,955	16,293.3	2835	16,293.3	2777
3rd	7,195,878	20,134	3,130,797	392,860	3,130,797	281,778	7913.5	1152	7913.5	1630
Max	31,484,048	2,552,975	14,855,260	1,629,179	14,855,260	1,083,991	1,219,545	159,005	1,219,545	204,963

Met = metagenomics, con = host contamination, pair = paired reads, unpair = unpaired reads

### *Detection* C. difficile *colonization*

All samples were tested for *C. difficile* toxin genes to determine *C. difficile* colonization. This was done using real-time polymerase-chain reaction with AdvanSure RT-PCR kit (LG Life Science) for the simultaneous detection of tcdA and tcdB genes. The primer pairs used were

NK9-NK11 for the repetitive domain of the tcdA gene and NK104-NK105 for the tcdB gene. This method is based on TaqMan technology and has been shown to have very good test characteristics with 100% sensitivity and 98.3% specificity.^92^ We used the SLAN RTPCR detection system (LG Life Science) according to the manufacturer’s instructions.^92^ Each sample needed to be positive for both tcdA and tcdB genes to then be categorized as positive for *C. difficile*. Estimation of *C. difficile* colonization using qPCR instead than directly from shotgun metagenomics has been used several times previously by us and others^93,94^ and it allows to determine with better resolution the presence of this bacterium which is usually found at very low abundance in the GI tract (<1%) and could be undetected from metagenomic sequencing.

### *Definition of outcome of* C. difficile *colonization*

We defined “*C. difficile* colonization” as the detection of *C. difficile* toxin genes A or B using real-time polymerase-chain reaction in the absence of any diarrheal symptoms 4 weeks pre- and post- sample collection. We further divided *C. difficile* colonization into two categories. The first was elders with only one sample testing positive (one-time) while the second was if the elder had multiple samples positive (multiple). No elders in this study had diarrhea at any point.

### Statistical and computational analysis

Multivariable Logistic Regression Modeling. We first used multivariable logistic regression analysis to test whether clinical variables alone were associated with *C. difficile* colonization. To select the set of covariates for the multivariable model, we selected any covariates with a *p* < .20 from an unadjusted bivariate analysis. We ran two models, first with the outcome of any colonization time point and then again with the outcome of multiple colonization time points. We included all acid-reducing medications rather than proton pump inhibitors alone in the model given that both were significantly associated with the outcome.

Descriptive Microbiome Analysis. To determine similarity in microbiome samples among the NH elders and to associate microbiome features to *C. difficile* colonization level, we started by performing traditional unsupervised correspondence analysis, including Principal Component Analysis and t-Distributed Stochastic Neighbor Embedding. We used functions within the R packages *phyloseq, vegan*, and *Rtsne* to perform the descriptive microbiome analysis (see available code at https://gitlab.com/vanni-bucci/2020_cdad_nh_paper).

Machine Learning Analysis. As most of the signal from the unsupervised analysis was accounted by inter-individual variability, we then decided to run supervised machine learning models that account for the repeated sampling nature of our study design. We therefore run mixed-effect random forest modeling to predict the abundance of every detected microbiota species as a function of clinical covariates and level of *C. difficile* colonization in each individual (see results). As reported in the results we used the Mixed-Effect-Random-Forest (MERF) routine from R package *LongituRF*^95^ to fit the model $X_{s,i\;}=\;f\left(CC\right)+1|\;ID$, where (1) X is the relative abundance of species s in sample i, (2) f is a general non-linear function (the random forest) applied to the Clinical Covariates (CC) as fixed effects (which includes *C. difficile* prevalence as ordinal variable 0,1,2) and (3) 1|ID corresponds to the random effect to account for multiple samples from the same patient. We used permutated importance analysis to assess the significance of the clinical predictors in associating with each microbiota species abundance.

To perform the within-patient analysis (e.g., differentiating positive and negative samples for elders that switch from negative to positive and vice-versa) we first calculated Shannon diversity and performed linear-mixed effect modeling as Diversity=SamplePositivity+1|ID. To classify samples as *C. difficile* positive vs negative, we applied Mixed-Effect Random Forest Classification. Permutated Importance Analysis was used to determine species significantly associated with sample positivity.

## Supplementary Material

Supplemental Material

## Data Availability

Sequences have been deposited in the NCBI SRA under accession PRJNA529586. Processed data, metadata and code to reproduce analysis in the paper is available at https://gitlab.com/vanni-bucci/2020_cdad_nh_paper.

## References

[1] Hall AJ, Curns AT, McDonald LC, Parashar UD, Lopman BA. The roles of Clostridium difficile and norovirus among gastroenteritis-associated deaths in the United States, 1999-2007. Clin Infect Dis: Infect Dis Soc. 2012;55(2):216–15. doi:10.1093/cid/cis386. 22491338

[2] Centers for Disease, C. a. P. Making health care safer: stopping C. difficile infections. https://www.cdc.gov/VitalSigns/Hai/StoppingCdifficile/ Accessed on 4**/**6**/**2020 (7 25, 2019).

[3] Lessa FC, Mu Y, Bamberg WM, Beldavs ZG, Dumyati GK, Dunn JR, Farley MM, Holzbauer SM, Meek JI, Phipps EC, et al. Burden of clostridium difficile infection in the United States. N Engl J Med. 2015;372(9):825–834. doi:10.1056/NEJMoa1408913. 25714160 PMC10966662

[4] Keller JM, Surawicz CM. clostridium difficile infection in the elderly. Clin Geriatr Med. 2014;30(1):79–93. doi:10.1016/j.cger.2013.10.008. 24267604

[5] McDonald LC, Owings M, Jernigan DB. clostridium difficile infection in patients discharged from US short-stay hospitals, 1996–2003. Emerg Infect Dis. 2006;12(3):409–415. doi:10.3201/eid1205.051064. 16704777 PMC3291455

[6] Loo VG, Bourgault A-M, Poirier L, Lamothe F, Michaud S, Turgeon N, Toye B, Beaudoin A, Frost EH, Gilca R, et al. Host and pathogen factors for clostridium difficile infection and Colonization. N Engl J Med. 2011;365(18):1693–1703. doi:10.1056/NEJMoa1012413. 22047560

[7] Abou Chakra CN, Pepin J, Sirard S, Valiquette L. Risk factors for recurrence, complications and mortality in Clostridium difficile infection: a systematic review. PloS One. 2014;9(6):e98400. doi:10.1371/journal.pone.0098400.g001. 24897375 PMC4045753

[8] Garg S, et al. Epidemiology of Clostridium difficile-associated disease (CDAD): a shift from hospital-acquired infection to long-term care facility-based infection. Dig Dis Sci. 2013;58(12):3407–3412. doi:10.1007/s10620-013-2848-x. 24154638

[9] Campbell RJ, et al. clostridium difficile infection in Ohio hospitals and nursing homes during 2006. J Soc Hosp Epiderm. 2009;30(6):526–533. doi:10.1086/597507. 19419272

[10] Kim JH, Toy D, Muder RR. clostridium difficile infection in a long-term care facility: hospital-associated illness compared with long-term care-associated illness. J Soc Hosp Epiderm. 2011;32(7):656–660. doi:10.1086/660767. 21666395

[11] Montoya A, Mody L. Common infections in nursing homes: a review of current issues and challenges. Aging Health. 2011;7(6):889–899. doi:10.2217/AHE.11.80. 23264804 PMC3526889

[12] Loo VG. Environmental interventions to control clostridium difficile. Infect Dis Clin North Am. 2015;29(1):83–91. doi:10.1016/j.idc.2014.11.006. 25573675

[13] Morgan XC, Huttenhower C. Chapter 12: human microbiome analysis. PLoS Comput Biol. 2012;8(12):e1002808. doi:10.1371/journal.pcbi.1002808. 23300406 PMC3531975

[14] Rea MC, et al. clostridium difficile carriage in elderly subjects and associated changes in the intestinal microbiota. J Clin Microbiol. 2012;50(3):867–875. doi:10.1128/JCM.05176-11. 22162545 PMC3295116

[15] Hopkins MJ, Macfarlane GT. Changes in predominant bacterial populations in human faeces with age and with clostridium difficile infection. J. Med. Microbiol. 2002;51(5):448–454. doi:10.1099/0022-1317-51-5-448. 11990498

[16] Claesson MJ, *et al*. Gut microbiota composition correlates with diet and health in the elderly. Nature. 2012;488(7410):178–184. doi:10.1038/nature11319. 22797518

[17] Salazar N, Valdes-Varela L, Gonzalez S, Gueimonde M, De Los Reyes-gavilan CG. Nutrition and the gut microbiome in the elderly. Gut Microbes. 2017;8(2):82–97. doi:10.1080/19490976.2016.1256525. 27808595 PMC5390822

[18] Petersen C, Round JL. Defining dysbiosis and its influence on host immunity and disease. Cell Microbiol. 2014;16(7):1024–1033. doi:10.1111/cmi.12308. 24798552 PMC4143175

[19] Wilkins LJ, Monga M, Miller AW. Defining dysbiosis for a cluster of chronic diseases. Sci Rep. 2019;9(1):12918. doi:10.1038/s41598-019-49452-y. 31501492 PMC6733864

[20] Haran JP, Bucci V, Dutta P, Ward D, McCormick B. The nursing home elder microbiome stability and associations with age, frailty, nutrition, and physical location. J Med Microbiol. 2018;67(1):40–51. doi:10.1099/jmm.0.000640. 29134939 PMC5884962

[21] Riggs MM, *et al*. Asymptomatic carriers are a potential source for transmission of epidemic and nonepidemic clostridium difficile strains among long-term care facility residents. Clin Infect Dis: Infect Dis Soc. 2007;45(8):992–998. doi:10.1086/521854. 17879913

[22] Morgan DJ, Leekha S, Croft L, Burnham CA, Johnson JK, Pineles L, Harris AD, Dubberke ER. The importance of colonization with clostridium difficile on infection and transmission. Curr Infect Dis Rep. 2015;17(9):499. doi:10.1007/s11908-015-0499-0. 26239132

[23] Clabots CR, Johnson S, Olson MM, Peterson LR, Gerding DN. Acquisition of clostridium difficile by hospitalized patients: evidence for colonized new admissions as a source of infection. J Infect Dis. 1992;166(3):561–567. doi:10.1093/infdis/166.3.561. 1323621

[24] Eyre DW, Griffiths D, Vaughan A, Golubchik T, Acharya M, O’Connor L, Crook DW, Walker AS, Peto TEA, *et al*. Asymptomatic clostridium difficile colonisation and onward transmission. PloS One. 2013;8(11):e78445. doi:10.1371/journal.pone.0078445. 24265690 PMC3827041

[25] Truong DT, *et al*. MetaPhlAn2 for enhanced metagenomic taxonomic profiling. Nat Methods. 2015;12(10):902–903. doi:10.1038/nmeth.3589. 26418763

[26] Hajjem A, Bellavance F, Larocque D. Mixed-effects random forest for clustered data. J Stat Comput Simul. 2014;84(6):1313–1328. doi:10.1080/00949655.2012.741599.

[27] Haran JP, *et al*. Alzheimer’s disease microbiome is associated with dysregulation of the anti-inflammatory p-glycoprotein pathway. mBio. 2019;10(3). doi:10.1128/mBio.00632-19 PMC6509190 31064831

[28] Altmann A, Toloşi L, Sander O, Lengauer T. Permutation importance: a corrected feature importance measure. Bioinformatics. 2010;26(10):1340–1347. doi:10.1093/bioinformatics/btq134. 20385727

[29] Riviere A, Selak M, Lantin D, Leroy F, De Vuyst L. Bifidobacteria and Butyrate-producing colon bacteria: Importance and strategies for their stimulation in the human gut. Front Microbiol. 2016;7:979. doi:10.3389/fmicb.2016.00979. 27446020 PMC4923077

[30] Parker BJ, Wearsch PA, Veloo ACM, Rodriguez-Palacios A. The genus alistipes: gut bacteria with emerging implications to inflammation, cancer, and mental health. Front Immunol. 2020;11:906. doi:10.3389/fimmu.2020.00906. 32582143 PMC7296073

[31] Wang K, Liao M, Zhou N, Bao L, Ma K, Zheng Z, Wang Y, Liu C, Wang W, Wang J, *et al*. Parabacteroides distasonis alleviates obesity and metabolic dysfunctions via production of succinate and secondary bile acids. Cell Rep. 2019;26(1):222–235.e225. doi:10.1016/j.celrep.2018.12.028. 30605678

[32] Granado-Serrano AB, Martín-Garí M, Sánchez V, Riart Solans M, Berdún R, Ludwig IA, Rubió L, Vilaprinyó E, Portero-Otín M, Serrano JCE, *et al*. Faecal bacterial and short-chain fatty acids signature in hypercholesterolemia. Sci Rep. 2019;9(1):1772. doi:10.1038/s41598-019-38874-3. 30742005 PMC6370822

[33] Franzosa EA, McIver LJ, Rahnavard G, Thompson LR, Schirmer M, Weingart G, Lipson KS, Knight R, Caporaso JG, Segata N, *et al*. Species-level functional profiling of metagenomes and metatranscriptomes. Nat Methods. 2018;15(11):962–968. doi:10.1038/s41592-018-0176-y. 30377376 PMC6235447

[34] Jochum L, Stecher B. Label or concept - what is a pathobiont? Trends Microbiol. 2020;28(10):789–792. doi:10.1016/j.tim.2020.04.011. 32376073

[35] Zaidi MB, Estrada-García T. Shigella: a highly virulent and elusive pathogen. Curr Trop Med Rep. 2014;1(2):81–87. doi:10.1007/s40475-014-0019-6. 25110633 PMC4126259

[36] Wexler HM. Bacteroides: the good, the bad, and the nitty-gritty. Clin Microbiol Rev. 2007;20(4):593–621. doi:10.1128/CMR.00008-07. 17934076 PMC2176045

[37] Kaper JB, Nataro JP, Mobley HL. Pathogenic escherichia coli. Nat Rev Microbiol. 2004;2(2):123–140. doi:10.1038/nrmicro818. 15040260

[38] d’Hennezel E, Abubucker S, Murphy LO, Cullen TW. Total lipopolysaccharide from the human gut microbiome silences toll-like receptor signaling. mSystems. 2017;2(6). doi:10.1128/mSystems.00046-17. PMC5686520 29152585

[39] Feng Z, *et al*. A human stool-derived Bilophila wadsworthia strain caused systemic inflammation in specific-pathogen-free mice. Gut Pathog. 2017;9(59). doi:10.1186/s13099-017-0208-7 PMC5657053 29090023

[40] Davis-Richardson AG, Ardissone AN, Dias R, Simell V, Leonard MT, Kemppainen KM, Drew JC, Schatz D, Atkinson MA, Kolaczkowski B, *et al*. Bacteroides dorei dominates gut microbiome prior to autoimmunity in Finnish children at high risk for type 1 diabetes. Front Microbiol. 2014;5:678. doi:10.3389/fmicb.2014.00678. 25540641 PMC4261809

[41] Forbes JD, Van Domselaar G, Bernstein CN. the gut microbiota in immune-mediated inflammatory diseases. Front Microbiol. 2016;7:1081. doi:10.3389/fmicb.2016.01081. 27462309 PMC4939298

[42] Finegold SM, *et al*. Anaerofustis stercorihominis gen. nov., sp. nov., from human feces. Anaerobe. 2004;10(1):41–45. doi:10.1016/j.anaerobe.2003.10.002. 16701499

[43] Ruiz L, Margolles A, Sanchez B. Bile resistance mechanisms in lactobacillus and bifidobacterium. Front Microbiol. 2013;4:396. doi:10.3389/fmicb.2013.00396. 24399996 PMC3872040

[44] Vakili B, Fateh A, Asadzadeh Aghdaei H, Sotoodehnejadnematalahi F, Siadat SD. intestinal microbiota in elderly inpatients with Clostridioides difficile infection. Infect Drug Resist. 2020;13:2723–2731. doi:10.2147/idr.S262019. 32801806 PMC7415437

[45] Trifan A, *et al*. Proton pump inhibitors therapy and risk of clostridium difficile infection: systematic review and meta-analysis. World J Gastroenterol. 2017;23(35):6500–6515. doi:10.3748/wjg.v23.i35.6500. 29085200 PMC5643276

[46] Behar L, Chadwick D, Dunne A, Jones CI, Proctor C, Rajkumar C, Sharratt P, Stanley P, Whiley A, Wilks M, *et al*. Toxigenic clostridium difficile colonization among hospitalised adults; risk factors and impact on survival. J Infect. 2017;75(1):20–25. doi:10.1016/j.jinf.2017.04.006. 28435086 PMC5464213

[47] Antharam VC, *et al*. Intestinal dysbiosis and depletion of butyrogenic bacteria in clostridium difficile infection and nosocomial diarrhea. J Clin Microbiol. 2013;51(9):2884–2892. doi:10.1128/JCM.00845-13. 23804381 PMC3754663

[48] Fachi JL, Felipe JDS, Pral LP, Da Silva BK, Corrêa RO, De Andrade MCP, Da Fonseca DM, Basso PJ, Câmara NOS, De Sales E Souza ÉL, *et al*. Butyrate protects mice from clostridium difficile-induced colitis through an hif-1-dependent mechanism. Cell Rep. 2019;27(3):750–761 e757. doi:10.1016/j.celrep.2019.03.054. 30995474

[49] Takahashi K, Nishida A, Fujimoto T, Fujii M, Shioya M, Imaeda H, Inatomi O, Bamba S, Andoh A, Sugimoto M, *et al*. reduced abundance of butyrate-producing bacteria species in the fecal microbial community in crohn’s disease. Digestion. 2016;93(1):59–65. doi:10.1159/000441768. 26789999

[50] Gardiner BJ, *et al*. Clinical and microbiological characteristics of eggerthella lenta bacteremia. J Clin Microbiol. 2015;53(2):626–635. doi:10.1128/JCM.02926-14. 25520446 PMC4298500

[51] Grinblat J, Weiss A, Grosman B, Dicker D, Beloosesky Y. Diarrhea in elderly patients due to clostridium difficile associated with salmonella and shigella infection. Arch Gerontol Geriatr. 2004;39(3):277–282. doi:10.1016/j.archger.2004.04.066. 15381346

[52] Falsen E, Kaijser B, Nehls L, Nygren B, Svedhem A. clostridium difficilein relation to enteric bacterial pathogens. J Clin Microbiol. 1980;12(3):297–300. doi:10.1128/JCM.12.3.297-300.1980. 7217331 PMC273578

[53] Rizzatti G, Lopetuso LR, Gibiino G, Binda C, Gasbarrini A. Proteobacteria: A common factor in human diseases. Biomed Res Int. 2017;2017:9351507. doi:10.1155/2017/9351507. 29230419 PMC5688358

[54] Kedia S, Ghosh TS, Jain S, Desigamani A, Kumar A, Gupta V, Bopanna S, Yadav DP, Goyal S, Makharia G, *et al*. Tu1792 – gut microbiome diversity in acute severe colitis is distinct from mild to moderate ulcerative colitis. Gastroenterology. 2019;156(6):S-1125-S-1126. doi:10.1016/s0016-5085(19)39778-1. 32870508

[55] Bercik P, Park AJ, Sinclair D, Khoshdel A, Lu J, Huang X, Deng Y, Blennerhassett PA, Fahnestock M, Moine D, *et al*. The anxiolytic effect of Bifidobacterium longum NCC3001 involves vagal pathways for gut-brain communication. J Eur Gastroint Motil Soc. 2011;23(12):1132–1139. doi:10.1111/j.1365-2982.2011.01796.x. PMC3413724 21988661

[56] Morgan XC, Tickle TL, Sokol H, Gevers D, Devaney KL, Ward DV, Reyes JA, Shah SA, LeLeiko N, Snapper SB, *et al*. Dysfunction of the intestinal microbiome in inflammatory bowel disease and treatment. Genome Biol. 2012;13(9):2–18. doi:10.1186/gb-2012-13-9-r79. PMC3506950 23013615

[57] Buffie CG, Bucci V, Stein RR, McKenney PT, Ling L, Gobourne A, No D, Liu H, Kinnebrew M, Viale A, *et al*. Precision microbiome reconstitution restores bile acid mediated resistance to clostridium difficile. Nature. 2015;517(7533):205–208. doi:10.1038/nature13828. 25337874 PMC4354891

[58] Ibrahim SA, Bezkorovainy A. Survival of bifidobacteria in the presence of bile salt. J Sci Food Agric. 1993;62(4):351–354. doi:10.1002/jsfa.2740620407.

[59] Mavromatis K, Stackebrandt E, Munk C, Lapidus A, Nolan M, Lucas S, Hammon N, Deshpande S, Cheng J-F, Tapia R, *et al*. Complete genome sequence of the bile-resistant pigment-producing anaerobe Alistipes finegoldii type strain (AHN2437(T)). Stand Genomic Sci. 2013;8(1):26–36. doi:10.4056/sigs.3527032. 23961309 PMC3739176

[60] Devkota S, Wang Y, Musch MW, Leone V, Fehlner-Peach H, Nadimpalli A, Antonopoulos DA, Jabri B, Chang EB. Dietary-fat-induced taurocholic acid promotes pathobiont expansion and colitis in Il10-/- mice. Nature. 2012;487(7405):104–108. doi:10.1038/nature11225. 22722865 PMC3393783

[61] Sistrunk JR, Nickerson KP, Chanin RB, Rasko DA, Faherty CS. Survival of the fittest: how bacterial pathogens utilize bile to enhance infection. Clin Microbiol Rev. 2016;29(4):819–836. doi:10.1128/CMR.00031-16. 27464994 PMC5010752

[62] Sorg JA, Sonenshein AL. Bile salts and glycine as cogerminants for clostridium difficile spores. J Bacteriol. 2008;190(7):2505–2512. doi:10.1128/JB.01765-07. 18245298 PMC2293200

[63] Linsky A, Gupta K, Lawler EV, Fonda JR, Hermos JA. proton pump inhibitors and risk for recurrent clostridium difficile infection. Arch Intern Med. 2010;170(9):772–778. doi:10.1001/archinternmed.2010.73. 20458084

[64] Kim JW. Proton pump inhibitors as a risk factor for recurrence of clostridium-difficile-associated diarrhea. World J of Gastroenterology. 2010;16(28):3573. doi:10.3748/wjg.v16.i28.3573. PMC2909558 20653067

[65] Le Bastard Q, Al-Ghalith GA, Grégoire M, Chapelet G, Javaudin F, Dailly E, Batard E, Knights D, Montassier E. Systematic review: human gut dysbiosis induced by non-antibiotic prescription medications. Aliment Pharmacol Ther. 2018;47(3):332–345. doi:10.1111/apt.14451. 29205415

[66] Imhann F, Bonder MJ, Vich Vila A, Fu J, Mujagic Z, Vork L, Tigchelaar EF, Jankipersadsing SA, Cenit MC, Harmsen HJM, *et al*. Proton pump inhibitors affect the gut microbiome. Gut. 2016;65(5):740–748. doi:10.1136/gutjnl2015-310376. 26657899 PMC4853569

[67] Freedberg DE, oussaint NC, Chen SP, Ratner AJ, Whittier S, Wang TC, Wang HH, Abrams JA. Proton pump inhibitors alter specific taxa in the human gastrointestinal microbiome: a crossover trial. Gastroenterology. 2015;149(4):883–885 e889. doi:10.1053/j.gastro.2015.06.043. 26164495 PMC4584196

[68] Tomkovich S, Lesniak NA, Li Y, Bishop L, Fitzgerald MJ, Schloss PD. The proton pump inhibitor omeprazole does not promote clostridioides difficile colonization in a murine model. mSphere. 2019;4(6). doi:10.1128/mSphere.00693-19 PMC6887860 31748246

[69] Sokol H, Pigneur B, Watterlot L, Lakhdari O, Bermúdez-Humarán LG, Gratadoux JJ, Blugeon S, Bridonneau C, Furet JP, Corthier G, *et al*. Faecalibacterium prausnitzii is an anti-inflammatory commensal bacterium identified by gut microbiota analysis of crohn disease patients. *Proceedings of the National Academy of Sciences of the United States of America* 2008;105:16731–16736. doi:10.1073/pnas.0804812105. PMC2575488 18936492

[70] Martín R, Miquel S, Benevides L, Bridonneau C, Robert V, Hudault S, Chain F, Berteau O, Azevedo V, Chatel JM, *et al*. functional characterization of novel faecalibacterium prausnitzii strains isolated from healthy volunteers: a step forward in the use of f. prausnitzii as a next-generation probiotic. Front Microbiol. 2017;8:1226. doi:10.3389/fmicb.2017.01226. 28713353 PMC5492426

[71] Bien J, Palagani V, Bozko P. The intestinal microbiota dysbiosis and clostridium difficile infection: is there a relationship with inflammatory bowel disease? Therap Adv Gastroenterol. 2013;6(1):53–68. doi:10.1177/1756283x12454590. PMC3539291 23320050

[72] Schaffler H, Breitruck A. clostridium difficile - from colonization to infection. Front Microbiol. 2018;9:646. doi:10.3389/fmicb.2018.00646. 29692762 PMC5902504

[73] Vincent C, Stephens DA, Loo VG, Edens TJ, Behr MA, Dewar K, Manges AR. Reductions in intestinal Clostridiales precede the development of nosocomial Clostridium difficile infection. Microbiome. 2013;1(18):18. 10.1186/2049-2618-1-18. 24450844 PMC3971611

[74] Schubert AM, Rogers MA, Ring C, Mogle J, Petrosino JP, Young VB, Aronoff DM, Schloss PD. Microbiome data distinguish patients with Clostridium difficile infection and non-C. difficile-associated diarrhea from healthy controls. mBio. 2014;5(3):e01021–01014. doi:10.1128/mBio.01021-14. 24803517 PMC4010826

[75] Shankar V, Hamilton MJ, Khoruts A, Kilburn A, Unno T, Paliy O, Sadowsky MJ. Species and genus level resolution analysis of gut microbiota in clostridium difficile patients following fecal microbiota transplantation. Microbiome. 2014;2(13):13. doi:10.1186/2049-2618-2-13. 24855561 PMC4030581

[76] Skraban J, Dzeroski S, Zenko B, Mongus D, Gangl S, Rupnik M. Gut microbiota patterns associated with colonization of different clostridium difficile ribotypes. PloS One. 2013;8(2):e58005. doi:10.1371/journal.pone.0058005. 23469128 PMC3585249

[77] Jeste DV, *Palmer BW, Appelbaum PS, Golshan S, Glorioso D, Dunn LB, Kim K, Meeks T, Kraemer HC*. A new brief instrument for assessing decisional capacity for clinical research. Arch Gen Psychiatry. 2007;64(8):966–974. doi:10.1001/archpsyc.64.8.966. 17679641

[78] Masnoon N, Shakib S, Kalisch-Ellett L, Caughey GE. What is polypharmacy? A systematic review of definitions. BMC Geriatr. 2017;17(1):230. doi:10.1186/s12877-017-0621-2. 29017448 PMC5635569

[79] Ticinesi A, Milani C, Lauretani F, Nouvenne A, Mancabelli L, Lugli GA, Turroni F, Duranti S, Mangifesta M, Viappiani A, *et al*. Gut microbiota composition is associated with polypharmacy in elderly hospitalized patients. Sci Rep. 2017;7(1):11102. doi:10.1038/s41598-017-10734-y. 28894183 PMC5593887

[80] Charlson ME, Pompei P, Ales KL, MacKenzie CR. A new method of classifying prognostic comorbidities in longitudinal studies: development and validation. J Chronic Dis. 1987;40(5):373–383. doi:10.1016/0021-9681(87)90171-8. 3558716

[81] Quan H, Li B, Couris CM, Fushimi K, Graham P, Hider P, Januel JM, Sundararajan V. Updating and validating the Charlson comorbidity index and score for risk adjustment in hospital discharge abstracts using data from 6 countries. Am J Epidemiol. 2011;173(6):676–682. doi:10.1093/aje/kwq433. 21330339

[82] Austin SR, Wong YN, Uzzo RG, Beck JR, Egleston BL. Why summary comorbidity measures such as the charlson comorbidity index and elixhauser score work. Med Care. 2015;53(9):e65–72. doi:10.1097/MLR.0b013e318297429c. 23703645 PMC3818341

[83] Rockwood K, Song X, MacKnight C, Bergman H, Hogan DB, McDowell I, Mitnitski A. A global clinical measure of fitness and frailty in elderly people. Can Med Assoc J. 2005;173(5):489–495. doi:10.1503/cmaj.050051. 16129869 PMC1188185

[84] Jackson MA, Jeffery IB, Beaumont M, Bell JT, Clark AG, Ley RE, O'Toole PW, Spector TD, Steves CJ. Signatures of early frailty in the gut microbiota. Genome Med. 2016;8(8). doi:10.1186/s13073-016-0262-7 PMC4731918 26822992

[85] Milani C, Ticinesi A, Gerritsen J, Nouvenne A, Lugli GA, Mancabelli L, Turroni F, Duranti S, Mangifesta M, Viappiani A, *et al*. Gut microbiota composition and clostridium difficile infection in hospitalized elderly individuals: a metagenomic study. Sci Rep. 2016;6(1):25945. doi:10.1038/srep25945. 27166072 PMC4863157

[86] Rubenstein LZ, Harker JO, Salvà A, Guigoz Y, Bruno Vellas B. Screening for undernutrition in geriatric practice: developing the short-form mini-nutritional assessment (MNA-SF). J Gerontol A Biol Sci Med Sci. 2001;56A(6):M366–372. doi:10.1093/gerona/56.6.M366. 11382797

[87] Saarela RK, Lindroos E, Soini H, Hiltunen K, Muurinen S, Suominen MH, Pitkälä KH. Dentition, nutritional status and adequacy of dietary intake among older residents in assisted living facilities. Gerodontology. 2014. doi:10.1111/ger.12144. 25163661

[88] Guigoz Y. the mini nutritional assessment (MNA) review of the literature–What does it tell us? J Nutr Health Aging. 2006;10:485–487. 17183419

[89] Bolger AM, Lohse M, Usadel B. Trimmomatic: A flexible trimmer for illumina sequence data. Bioinformatics. 2014;30(15):2114–2120. doi:10.1093/bioinformatics/btu170. 24695404 PMC4103590

[90] Langmead B, Salzberg SL. Fast gapped-read alignment with bowtie 2. Nat Methods. 2012;9(4):1–3. doi:10.1038/nmeth.1923. 22388286 PMC3322381

[91] Truong DT, Franzosa EA, Tickle TL, Scholz M, Weingart G, Pasolli E, Tett A, Huttenhower C, Segata N. MetaPhlAn2 for enhanced metagenomic taxonomic profiling. Nat Methods. 2015;12(10):902–903. doi:10.1038/nmeth.3589. 26418763

[92] Kim H, Jeong SH, Kim M, Lee Y, Lee K. Detection of clostridium difficile toxin A/B genes by multiplex real-time PCR for the diagnosis of C. difficile infection. J Med Microbiol. 2012;61(2):274–277. doi:10.1099/jmm.0.035618-0. 21959205

[93] Buffie CG, Jarchum I, Equinda M, Lipuma L, Gobourne A, Viale A, Ubeda C, Xavier J, Pamer EG. Profound alterations of intestinal microbiota following a single dose of clindamycin results in sustained susceptibility to clostridium difficile-induced colitis. Infect Immun. 2012;80(1):62–73. doi:10.1128/IAI.05496-11. 22006564 PMC3255689

[94] Matsuda K, Tsuji H, Asahara T, Takahashi T, Kubota H, Nagata S, Yamashiro Y, Nomoto K. Sensitive quantification of clostridium difficile cells by reverse transcription-quantitative PCR targeting rRNA molecules. Appl Environ Microbiol. 2012;78(15):5111–5118. doi:10.1128/aem.07990-11. 22582062 PMC3416433

[95] Capitaine L, Genuer R, Thiébaut R. Random forests for high-dimensional longitudinal data. Stat Methods Med Res. 2021. doi:10.1177/0962280220946080 32772626

