# Supplementary material for: The high prevalence of Clostridioides difficile among nursing home elders associates with a dysbiotic microbiome
Source: Gut Microbes. 2021 Mar 25;13(1):1897209. doi: 10.1080/19490976.2021.1897209 (PMC8007149; doi:10.1080/19490976.2021.1897209)
Supplement: Supplemental Material [file KGMI_A_1897209_SM1931.zip › Supplementary information/Figure captions.docx]

**Figure S1:** t-distributed stochastic neighbor embedding analysis show sample clustering based on patient ID.

**Figure S2:** Pathways significantly associated with *C. difficile* prevalence from mixed-effect random forest modeling identifying bacteria metabolic pathways (BH adjusted < 0.05) using humann2. We report barplots showing the average +/- standard deviation of the abundance of CD-colonization significantly associated metabolic pathways in patients Never, Once or with Multiple samples colonized by *C. difficile*.

**Figure S3:** C. difficile positivity analysis. A) Shannon diversity comparison of sample negative vs positive for C. difficile for 66 elders presenting both positive and negative samples. Linear mixed-effect modeling was performed (See Text). B) Species found significantly associated with C. difficile positivity from Mixed-Effect Random Forest Classification.
