# Supplementary figures and images for: The high prevalence of Clostridioides difficile among nursing home elders associates with a dysbiotic microbiome
Source: Gut Microbes. 2021 Mar 25;13(1):1897209. doi: 10.1080/19490976.2021.1897209 (PMC8007149; doi:10.1080/19490976.2021.1897209)

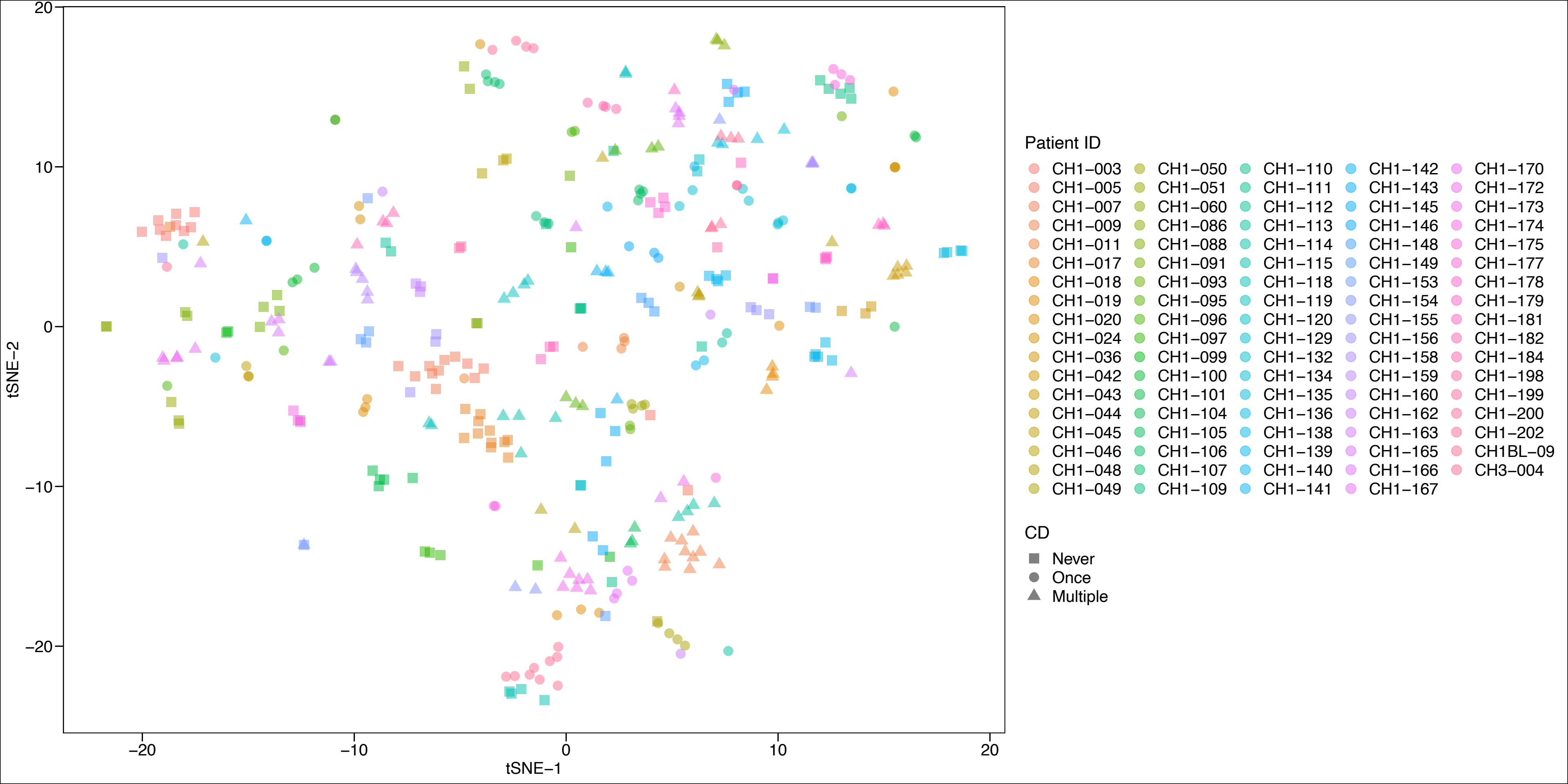

Supplement: Supplemental Material [file KGMI_A_1897209_SM1931.zip › Supplementary information/figure_S1.pdf]

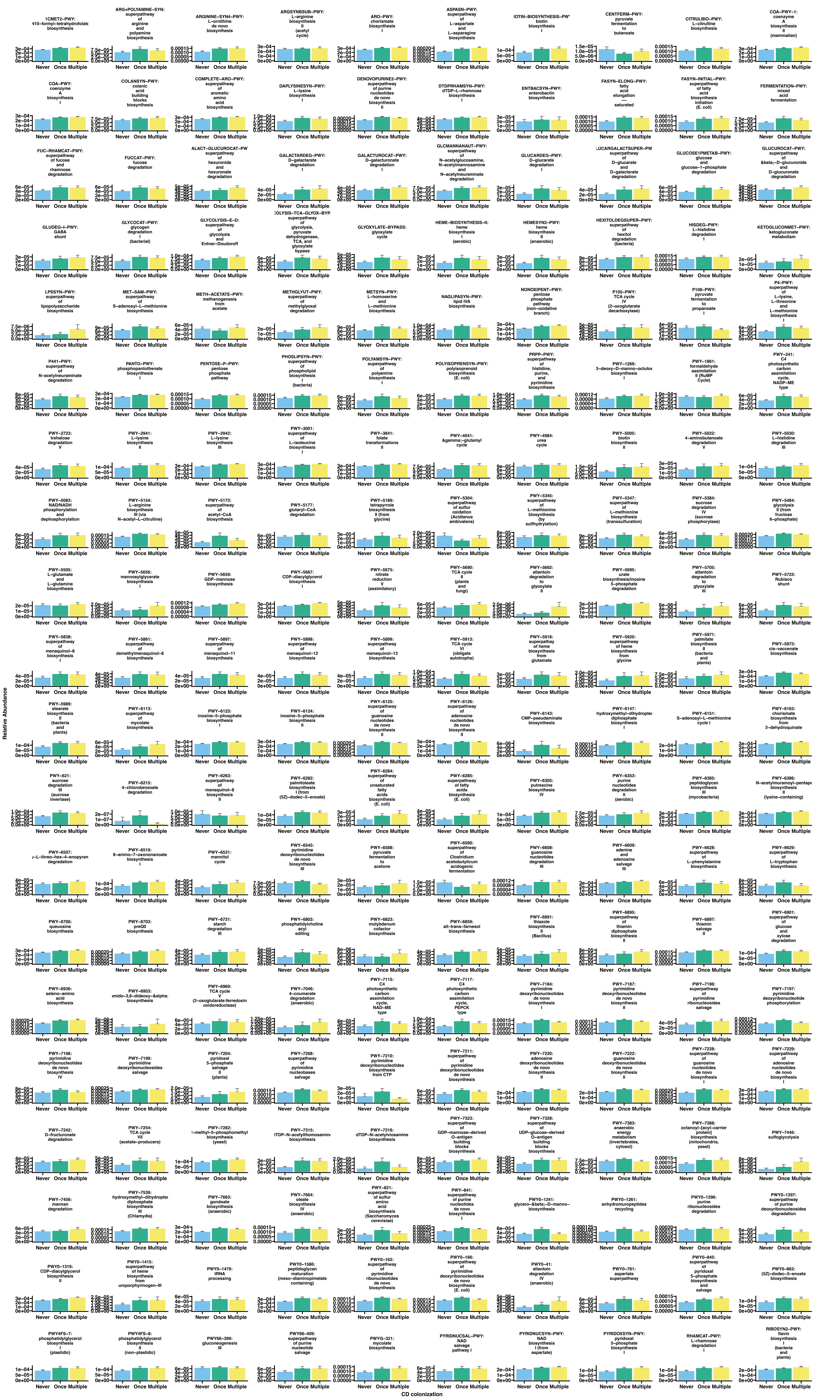

Supplement: Supplemental Material [file KGMI_A_1897209_SM1931.zip › Supplementary information/figure_S2.pdf]

A

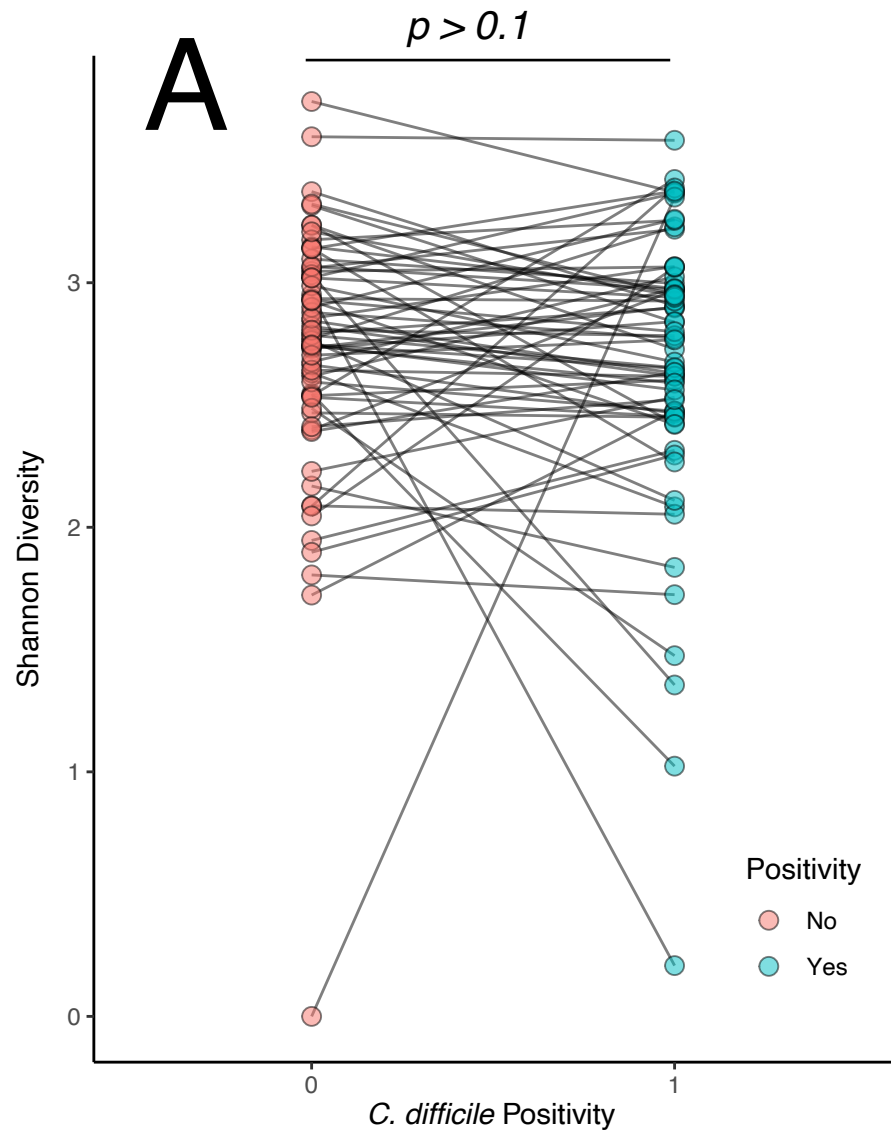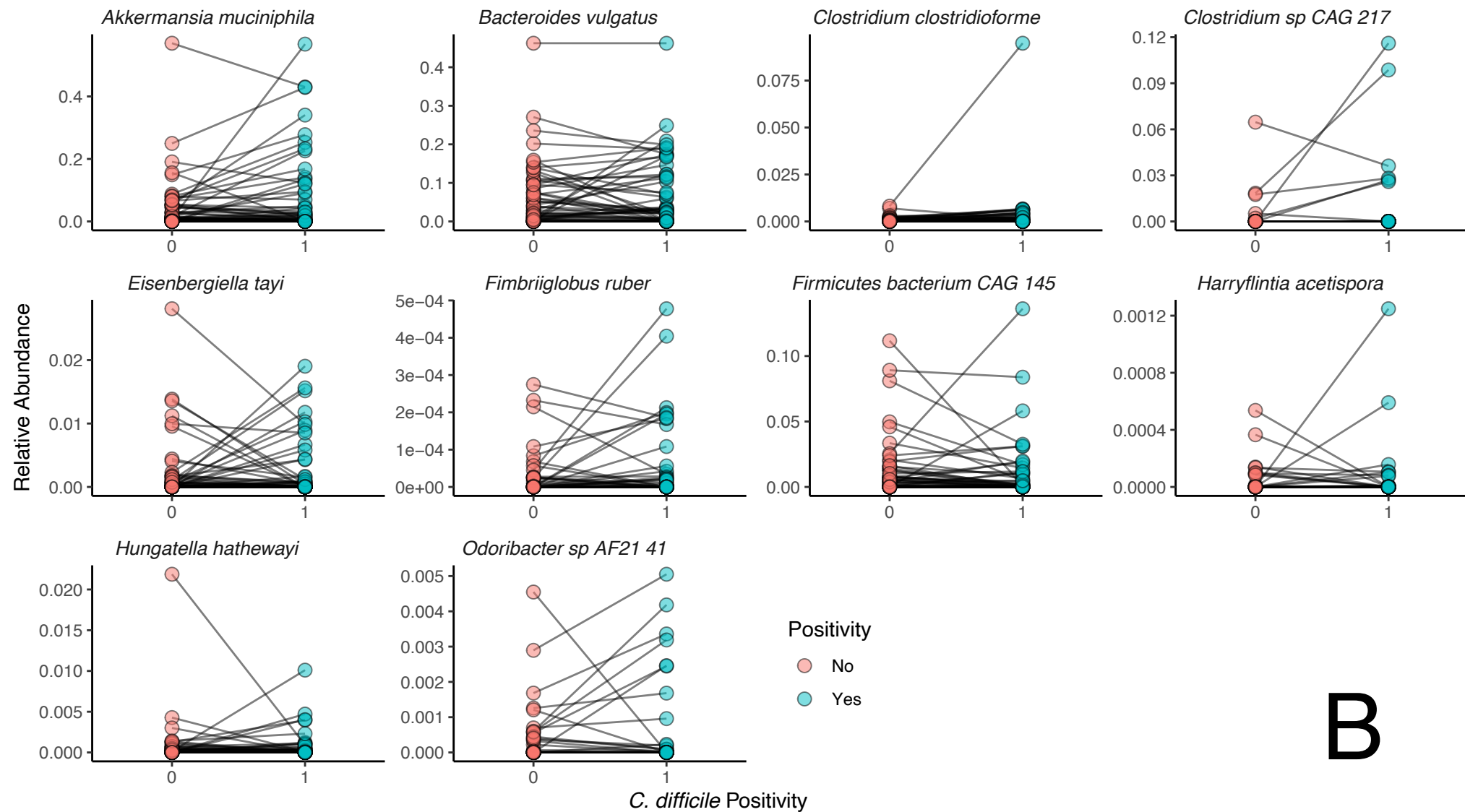

B

Supplement: Supplemental Material [file KGMI_A_1897209_SM1931.zip › Supplementary information/figure_S3.pdf]
